# Antitumor Drugs and Their Targets

**DOI:** 10.3390/molecules25235776

**Published:** 2020-12-07

**Authors:** Zlatko Dembic

**Affiliations:** Molecular Genetics Laboratory, Department of Oral Biology, Faculty of Dentistry, University of Oslo, 0316 Oslo, Norway; zlatko.dembic@odont.uio.no

**Keywords:** cancer, chemotherapy, biologics, immunotherapy, cancer hallmarks, immune system, immune checkpoint

## Abstract

Through novel methodologies, including both basic and clinical research, progress has been made in the therapy of solid cancer. Recent innovations in anticancer therapies, including immune checkpoint inhibitor biologics, therapeutic vaccines, small drugs, and CAR-T cell injections, mark a new epoch in cancer research, already known for faster (epi-)genomics, transcriptomics, and proteomics. As the long-sought after personalization of cancer therapies comes to fruition, the need to evaluate all current therapeutic possibilities and select the best for each patient is of paramount importance. This is a novel task for medical care that deserves prominence in therapeutic considerations in the future. This is because cancer is a complex genetic disease. In its deadly form, metastatic cancer, it includes altered genes (and their regulators) that encode ten hallmarks of cancer-independent growth, dodging apoptosis, immortalization, multidrug resistance, neovascularization, invasiveness, genome instability, inflammation, deregulation of metabolism, and avoidance of destruction by the immune system. These factors have been known targets for many anticancer drugs and treatments, and their modulation is a therapeutic goal, with the hope of rendering solid cancer a chronic rather than deadly disease. In this article, the current therapeutic arsenal against cancers is reviewed with a focus on immunotherapies.

## 1. Introduction

Anticancer therapies aim to restrain the growth and spread of cancer. They can be divided in many ways. Traditionally, they are divided by the type of procedure, such as surgery, radiotherapy, chemotherapy, targeted therapy, immunotherapy, or cell therapy, but this distinction can sometimes be blurred because of combinatorial therapies, which are becoming increasingly attractive. It is the advent of immune checkpoint therapies that has alerted researchers to the possibility that cancer could soon become a chronic rather than a deadly disease. These therapies (which also include cell therapies like CAR-T cells) are currently being studied in over 3000 FDA-approved clinical studies (in phase 2 and 3) in various types of cancer as combinatorial therapies, with nearly all the tools that were previously available in the chemotherapy of cancer [1]. We need to understand the essence of immune checkpoint immunotherapies if we wish to improve them by using small molecules as anticancer drugs in combination with them. Essentially, this is the aim of this mini review. I will summarize the current state of therapies, illustrate historical perspectives of approved anticancer treatments and explain strategies aimed at discovering novel anticancer drugs.

All treatments can be divided according to the characteristics of the cancer which they target and, in so doing, either inhibit or interfere with it. We lack knowledge of all molecular pathways, their genes and the epigenetic elements involved in malignant tissue transformation, its invasive spreading and metastasis. However, we know enough to begin to construct an idea about the genetic and epigenetic landscapes of each and every tumor type. There are worldwide projects aiming to map the cancer genetics and epigenetics as well as their proteomic landscape. Such data can be found in databases such as the Cancer Genomics, created by the National Cancer Institute (NCI) in 1997. This is an online reference on normal, pre-cancerous and cancerous genomes and, in addition, offers tools for viewing and analyzing the data, including browsing the genes reported for various phases of tumor progression. Seminal publications by Hanahan and Weinberg [2,3] suggested that an eukaryotic cell can become malignant by accumulating at least one mutation in each of the ten characteristics (hallmarks) of cancer. We are now aware that cancer stem cells (CSC) can form by sequentially accumulating such changes over a long period of time (years), from which a cancer could develop. Importantly, although some are unique for each type of cancer, some changes can be common for many different cancer types. Hence, each of these cancer types might be treated by a combination therapy that targets common as well as specific cancer hallmarks.

## 2. Development of Cancer

**Figure 1:**
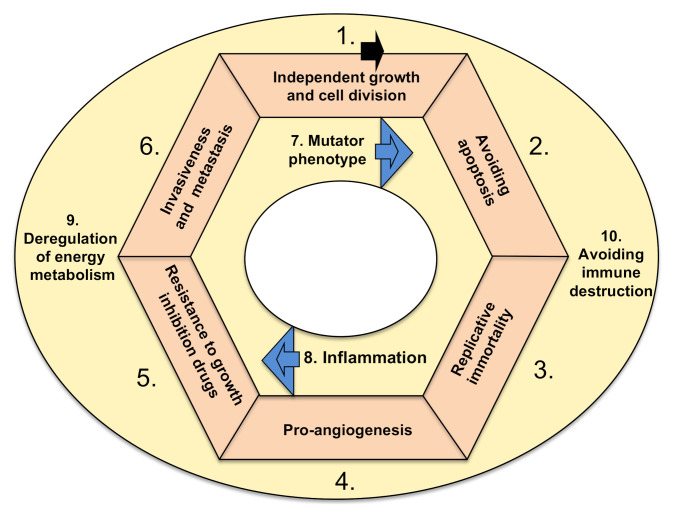
Hallmarks of cancer. The arrow denotes a possible order of new mutations occurring in cancer cells, forming a loop (modified, based on Hanahan and Weinberg [2]). Tumor accumulates mutations (or epigenetic hits) and acquires listed hallmarks. Hallmarks 7 and 8 are accelerating features [2]. Hallmarks 9 and 10 could occur anytime within the cycle, and I suggest that they are cancer-supporting characteristics.

There are ten basic functional characteristics of cancer, which are called hallmarks. In order for a tumor to become malignant (i.e., able to invade surrounding tissues and metastasize) and lead to a deadly disease, either genetic or epigenetic changes must take place in all hallmarks (Figure 1). There are exceptions to this rule, which are rare. Namely, benign tumors (i.e., glioma, if in the head) or those localized inconveniently, preventing the life-supporting feature of the tissue, could be also deadly.

The hallmarks are characterized by the following functional categories: (1) sustaining proliferative signaling, leading to independent growth, (2) resisting cell death (i.e., downregulation of apoptosis), (3) cellular immortalization (prolonging telomeres, enabling cell replication in perpetuity), (4) induction of angiogenesis, (5) resisting conventional therapies that include drugs as suppressors of growth, (6) invasive spreading with metastasis, (7) increased genetic/epigenetic instability (and attaining mutator phenotype), (8) tumor-promoting inflammation (as a growth-stimulating factor), (9) deregulation of energy metabolism in order to acquire more energy for growth (including metabolic shift from oxidative to anaerobic glycolysis) and (10) escaping destruction by the immune system [2]. The ten hallmarks are depicted in Figure 1. There is a hypothesis that various tissue types of cancer depend on particular combinations of genetic/epigenetic changes within the ten hallmarks. The drugs used in cancer therapies that can target and interfere with the ten hallmarks of cancer are elaborated below, firstly from the historic perspective of approval for therapy and secondly through their mode of action.

## 3. Cancer Stem Cells

Cancer is the expression used to describe an exceedingly complex group of diseases, caused by changes to the genes in one cell or a group of cells, resulting in altered protein–protein interactions. There are at least two major groups of hypotheses on how cancer can arise. One stipulates that it can arise from normal cells that undergo numerous gene mutations, epigenetic modifications, unrestrained signaling pathways and changes in the regulation of the microenvironment that follow the cycle of events depicted in Figure 1.

Alternatively, at some point during the changes within the genetic constellation of a particular cell in the affected tissue, a very small subpopulation of cancer cells can arise, called cancer stem cells, which then similarly follow a cycle of progression towards more malignant forms.

In the latter case, and in more detail, cancer forms a distinct tumor microenvironment, in which cancer stem cells (CSC) are tumor-(re)generating cells. Cancer stem cells exhibit self-renewal and multilineage capacities. CSCs divide, and a part of their progeny differentiates, while some CSCs mutate further, evolving into more invasive kinds of CSCs until they spread to the surrounding tissue and ultimately metastasize [4,5]. Thus, in solid cancers, other stromal cells like cancer fibroblasts are derived in major part from cancer stem cell. However, part of the cancer includes normal cells that have migrated there, like tumor-infiltrating lymphocytes, macrophages, vascular epithelium or fibroblasts [6]. Despite seeming normal, under the influence of cancer cells and the tumor microenvironment, cells from other parts of the body and outside the tumor mass begin to “behave” abnormally and perhaps might not have mutations in genes as CSCs in order to become a part of that particular cancer. Such could be the fate of many endothelial cells and fibroblasts, in general. We see also that lymphatic vessels can only be detected outside the tumor mass, probably because, inside solid tumors, they become “something else”. CSCs are considered resistant to chemotherapy or radiation, both of which can destroy other non-stem-cell tumor cells (and most cells from other parts of the body). CSCs are first described in myeloid leukemia [7,8] and thereafter in solid tumors [9,10]. CSCs’ roles were assessed in animal models [11] and by studying their transcriptional profiles [12]. CSCs resemble embryonic stem cells and induced pluripotent stem cells by expressing similar sets of transcription factors [13,14]. It is thought that CSCs are made by the accumulation of genetic/epigenetic changes until they achieve the ability to self-renew, produce differentiated progeny and develop resistance to therapy. Alternatively, CSCs could be formed by reprogramming from a differentiated cell of a tissue at some point in the life of an individual [15]. CSCs have a fundamental role in providing cancers with resistance to treatment, resurgence and metastasis. CSCs employ cell-signaling molecules in the Hedgehog, Wnt and Notch pathways, and drugs targeting these pathways showed encouraging results in experiments with multiple types of cancer [16,17]. Therefore, targeting CSCs could be a promising strategy for anticancer therapy [18].

## 4. Historical Perspective of Anticancer Therapies

Surgery is the oldest treatment for cancer, used over the last three millennia, albeit with varying success (reviewed in [19]). It was recorded that ovariectomy decreased the incidence of breast cancer in women in the second half of the 19th century, showing an influence of sex hormones on cancer development [20,21]. Soon after the discovery of X-rays by Wilhelm C. Röntgen (1895), physicians began using them to treat various lesions of the skin, including lupus, basal cell carcinoma and epithelioma [22]. In 1896, X-rays were reported to be used for the first time in cancer therapy, to treat breast cancer patients, by Emil H. Grubbe [23,24], and in 1899, they were used by Anton U. Sjøgren to treat epithelioma of the mouth [25]. The Nobel Prize for Physics was awarded in 1903 for the discovery of radioactivity by Henry Becquerel (1886) and radium by Pierre Curie and Marie Sklodowska-Curie (1898). The biologic effects of radium led the latter two to suggest in 1899 that radioactivity could be used in treating cancer patients [26,27]. In 1907, the “Kassabian S Medical Manual” in Philadelphia listed tumors treated by radiotherapy [28], some of which are still in use [27]. Decades later, at the International Congress of Oncology in Paris in 1922, French radiologist Henry Coutard presented cases when radiotherapy could be used without disastrous side effects to successfully treat cancers located in the buccal cavity, the larynx and pharynx—data that were published at a later date [29]. Together with Claudius Regaud, who showed that radiation fractionation could be used to treat several human cancers, reducing the side effects [30,31], these treatments marked the dawn of modern radiotherapy. In the 1940s, chemotherapeutics started to be developed and used in cancer treatment [32]. They were derived from toxins made for chemical warfare, such as nitrogen mustards and antifolate drugs [33]. Gustav Lindskog treated a patient with lymphosarcoma (which was X-ray resistant) with nitrogen mustard in 1942 [34]. This was the first evidence that chemotherapy can cause cancer regression.

Thus, the first chemotherapy approved by the U.S. Food and Drug Administration (FDA [35]) was nitrogen mustard mustine in 1949 [36]. (Underlining of drugs in text indicates their mentioning in Tables and/or Figures). Soon thereafter, other nitrogen mustards were developed, and they included cyclophosphamide, chlorambucil, uramustine, melphalan and bendamustine [37]. Although mustine was discontinued as a cancer therapy because of its extensive toxicity, others have gained therapeutic roles over time. Therefore, it is important to consider the timeline of anticancer drug approvals until the present (starting with Table 1). Previously, many reviews have described this timeline, but only a few include the most recent approvals [19,38,39,40,41,42,43].

In 1953, two drugs, methotrexate and 6-mercaptopurine, were approved for treatment against cancer as they possess anti-metabolic activities that inhibit unwanted growth [44]. Inhibition of cancer cell proliferation and growth was the aim in these and the following several approved therapies, such as vinblastine (1961), isolated from Madagascar periwinkle in 1958 [45] (p. 157). In 1962, the drug 5-fluorouracil (5-FU) received the FDA’s approval [46], and it is, together with others, still in use in some combinatorial treatments. Since then, the list of chemotherapeutic anticancer agents has increased. They are listed in Table 2, together with the description of their antitumor effect.

The next chemotherapy was approved in 1964: this was melphalan [47] (Table 1 and Table 2), which is still in use and was recently (in 2020, as *Phelinun* by Adienne S.r.l. S.U.) approved by the European Medicines Agency (EMA, previously known as EMEA) for various malignancies (alone, or in combination with other cytotoxic medicinal products and/or total body irradiation) [48].

Then, 10 years passed until the next drug was approved; it was anthracycline antibiotic doxorubicin, isolated from *Streptomyces paucetius* bacteria [45] (p. 291) (Table 1 and Table 2) [49]. In 1975, dacarbazine was approved for the treatment of melanoma and Hodgkin’s lymphoma [50]. (Currently, the latter two are part of two anticancer drug regimens: ABVD (with bleomycin and vinblastine) in the treatment of Hodgkin’s lymphoma [51] and MAID (with mesna and ifosfamide) for sarcoma [52]).

Following the historical perspective (Table 1), in 1977, carmustine was approved for the palliative treatment of various brain tumors including glioblastoma multiforme [53,54]. In addition, tamoxifen, a novel therapeutic molecule that targeted estrogen synthesis, was approved for the treatment of breast cancer. Another 10 years passed, and ifosfamide was approved, a drug that, currently, is usually given as an anticancer agent after other treatments have failed [55]. Then, carboplatin was approved as an anticancer drug in 1989 [56]. In 1991, paclitaxel was approved for advanced ovarian cancer and was the first of taxans, which were developed later as anticancer drugs [57]. In 1995, all-trans retinoic acid, or tretinoin, a drug related to vitamin A, was approved for the treatment of acute promyelocytic leukemia [58]. In 1996, topotecan [59] and irinotecan [60], drugs that are DNA *topoisomerase I* inhibitors (Table 2), were approved for therapies of metastatic ovarian and colorectal cancers, respectively (Table 1). In the same year, oxaliplatin was approved for the treatment of colorectal cancer in France [61]. Similarly, for breast cancers, the EMA approved the use of letrozole [62] as adjuvant therapy for early-stage breast cancer in postmenopausal women already treated for 5 years with tamoxifen. (Both therapies were 8 years ahead of their FDA approval). In the following year, 1997, the first biologic was approved as an anticancer drug for non-Hodgkin’s lymphoma (Table 1). This was monoclonal antibody anti-CD20, rituximab [63]. Recently, in 2017, the FDA granted approval to rituximab and hyaluronidase (Rituxan Hycela) for the treatment of patients with follicular lymphoma, diffuse large B cell lymphoma and chronic lymphocytic leukemia (CLL) [63].

The year of 1997 marks the dawn of the age of biologics, in terms of monoclonal antibodies interfering with various hallmarks of cancer. Already in 1998, trastuzumab (Herceptin), a monoclonal antibody specific for cancer cells that produce excessive oncoprotein HER2 (human epidermal growth factor receptor 2, also known as ErbB2), was approved in the treatment of metastatic breast cancer [64] (Table 1).

In 2006, an upgrade in therapy with trastuzumab was approved for use as an adjuvant to treat women with early-stage node-positive HER2-overexpressing breast cancer [65,66].

The next biologics were approved early in the 21st century, with humanized monoclonal antibody alemtuzumab (Campath1), which targets CD52 on B, T and NK cells and monocytes, in the therapy of chronic lymphocytic leukemia (2001) [67] (Table 1). The following two years witnessed the approval of the radionuclide-linked monoclonal antibodies ibritumomab tiuxetan (Zevalin; anti-CD20) [68] and tositumomab (Bexxar; anti-CD19) [69,70] to treat non-Hodgkin’s lymphoma (2002–3), indicating the beginning of antibody-targeted radiotherapy of cancer [71]. However, the marketing approval of the latter was discontinued in 2014 (by FDA) [72], as well as its orphan drug designation for the treatment of follicular lymphoma (awarded in 2003 by EMA) in 2015 [73], possibly due to a decline in usage, supply chain issues, high pricing or perhaps the emergence of non-radioactive competitors.

In 2004, two biologics, cetuximab (Erbitux) [74] and bevacizumab (Avastin) [75], were approved for therapy of metastatic colorectal cancer (Table 1). Cetuximab targets and inhibits signals from epidermal growth factor receptor (EGFR) expressed in some cancers, inducing cell arrest and apoptosis, and bevacizumab blocks the action of vascular endothelial growth factor (VEGF), thereby inhibiting angiogenesis.

In 2005, two drugs were approved by the FDA for treating breast cancer. They were aromatase inhibitors, anastrozole [76] (previously approved in Europe in 1995 [77]) and exemestane, approved as adjuvant therapy of hormone-receptor-positive early-stage breast cancer [78] (Table 1). In 2006, thalidomide [79] and lenalidomide [80] were approved for the treatment of multiple myeloma. In the same year, the FDA approved the first fully human monoclonal antibody for cancer therapy, namely panitumumab (Vectibix, Amgen), for the treatment of metastatic colorectal cancer [81].

In 2008, in Russia (Table 3), the first approval in the world was given to therapeutic vaccine oncophage for renal cell carcinoma in humans (using autologous tumor-derived heat shock protein gpg6) [82]. One year later, in 2009, the FDA approved cervarix, the prophylactic vaccine against two types of HPV (16 and 18) that cause around 70% cases of cervical cancer worldwide [83]. In 2010, the FDA approved the first therapeutic cancer vaccine, sipuleucel-T (Provenge), for castration-resistant prostate cancer [84].

The first immunotherapy, in *strictu senso*, meaning an inhibition of the regulation of the adaptive immune response against cancer, was approved in 2011. The EMA, Japan and the Australian Therapeutic Goods Administration (TGA) approved ipilimumab (Yervoy), an immune checkpoint inhibitor of CTLA-4, for the treatment of melanoma [85]. This was the first drug of any kind that showed extended survival in such patients. Comparably, the FDA approved ipilimumab for metastatic melanoma [86] and, in addition, vemurafenib (Zelboraf, Roche), a BRAF serine-threonine kinase inhibitor [87], and peginterferon alfa-2b (Sylatron) for the therapy of melanoma [88] (Table 3).

In the same year, in 2011, a drug-linked biologic conjugate was approved by the FDA to treat Hodgkin’s lymphoma and anaplastic large cell lymphoma (Table 3). It was brentuximabvedotin (Adcetris, Seattle Genetics, Inc., Bothell, WA, USA), a cytotoxic agent-linked chimeric mouse/human anti-human CD30 monoclonal antibody [89]. Brentuximab vedotin conjugate binds to CD30 and enters the targeted cell. Inside, its attached cargo, the synthetic microtubule disrupting agent, monomethyl auristatin E (MMAE), is released by the proteases, leading to cancer cell death [90]. In 2018, the EMA and FDA extended the approval to the treatment of patients with previously untreated classical Hodgkin’s lymphoma in combination with chemotherapy [91].

In 2014, the FDA approved blinatumomab (Blincyto, Amgen) for use in the treatment of B cell acute lymphoblastic leukemia (ALL) (Table 3). Blincyto is the first of a novel class of drugs called bispecific T cell engagers (BiTE). BiTE consist of two monoclonal antibodies joined together. While one end binds to a molecule on T cells, the other one binds to a molecule on cancer cells (CD19), facilitating its killing [92]. Presently, blinatumomab is also used for the treatment of relapsed or refractory B-ALL (since 2017), adult and pediatric patients with B-ALL who are in remission but have signs of minimal residual disease (since 2018) [93].

In 2014, the FDA also approved ramucirumab (Cyramza, Eli Lilly and Co), an anti-angiogenic biologic, to treat advanced stomach cancer and gastroesophageal junction adenocarcinoma [94].

In 2014, the FDA and Japan (and TGA, Health Canada and EMA in early 2015) approved two monoclonal antibodies directed against PD-1 molecule for surgically inoperative melanoma (Table 3). The monoclonal antibodies pembrolizumab (Keytruda, Merck) [95] and nivolumab (Opdivo, Bristol-Myers Squibb) [96] prevent the interaction of their target molecule (PD-1) with its ligands, thus acting as immune checkpoint inhibitors.

In 2014, the EMA (and the FDA in 2017) granted approval to a new CAR-T cell immunotherapy (Table 3), tisagenlecleucel (Kymriah, Novartis), which targets CD19, for the treatment of B-ALL that is refractory or has relapsed after at least two previous treatments (and in 2016 (the FDA in 2018) for refractory or relapsed large B-cell lymphoma) [97,98].

In 2017 (Table 4), approval was given to olaratumab (Lartruvo), a targeted antibody against the platelet-derived growth factor receptor alpha (PDGFRα), for patients with soft tissue sarcoma who were left without options to be treated by surgery and radiation [99]. Unfortunately, a phase 3 trial has found no prolongation of survival in patients with soft tissue sarcoma [100]. This led both the EMA and FDA (2019) to revoke their approval [101].

In the same year, the FDA granted approval to immune checkpoint inhibitors targeting the PD-1 pathway—durvalumab (Imfinzi, AstraZeneca) [102] and avelumab (Bavencio, EMD Serono, Inc. Rockland, MA, USA) [103], for advanced bladder cancer, and atezolizumab (Tecentriq, Genentech/Roche), for patients with metastatic, chemotherapy-resistant non-small cell lung cancer (NSCLC) [104] (Table 4 and Table 5).

Similarly, in 2017, approval was given to axicabtagene ciloleucel (Yescarta, Kite/Gilead) for the treatment of several types of non-Hodgkin’s large B cell lymphomas refractory or twice relapsed after previous systemic treatments [105].

An interesting antibody–drug conjugate that targets the CD33 molecule was granted approval in 2017 by the FDA. Monoclonal anti-CD33 conjugated to toxin-gemtuzumab ozogamicin (Mylotarg) was approved to treat patients with CD33-positive acute myeloid leukemia (AML) [106].

In 2018, the FDA granted approval for the treatment of the second most common type of skin cancer to a novel checkpoint inhibitor (that targets the PD-1 pathway), cemiplimab (Libtayo, Regeneron Pharmaceuticals) [107]. The approval was for the treatment of advanced forms of cutaneous squamous cell carcinoma. The EMA and the TGA also approved Libtayo in 2019 and 2020, respectively [107,108].

In 2019, the FDA approved pexidartinib (Turalio), a small-molecule immunomodulator that targets the cytokine “colony stimulating factor-1(CSF-1)” receptor pathway, to treat symptomatic tenosynovial giant cell tumor [109].

Additionally in 2019, a novel small-molecule inhibitor of apoptosis (via Bcl-2 inhibition) [110], Venetoclax (Venclexta, Venclycto), was approved by the FDA (EMA, in 2020) for the treatment of chronic lymphocytic leukemia (CLL), small lymphocytic lymphoma (SLL) and acute myeloid leukemia (AML) [111].

In 2020, the FDA approved brexucabtagene autoleucel (Tecartus), a CAR-T cell immunotherapy that targets the CD19 receptor, for the treatment of patients with relapsed or refractory mantle cell lymphoma [112].

In 2020, the human papilloma virus (HPV) prophylactic vaccine *Gardasil 9* (previously approved for cervical cancer prevention by the FDA in 2006 [113]) received extended approval for the prevention of HPV-related head and neck cancers [114].

The mode of action for the listed drugs in Table 1, Table 3 and Table 4 was assessed from various sources cited in the text. The cell cycle blockage assessments were based on the e-book by Dowd et al. (chapter “Anti-neoplastic drugs” p. 521) [115].

Notwithstanding these achievements, the therapies targeting immune checkpoints CTLA4 and PD-1 pathways signify a revolution in cancer treatment. There are currently (Table 5) seven approved biologics in this group: ipilimumab (Yervoy, 2011), pembrolizumab (Keytruda, 2014), nivolumab (Opdivo, 2014), durvalumab (Imfinzi, 2014), avelumab (Bavencio, 2014), atezolizumab (Tecentriq, 2014) and cemiplimab (Libtayo, 2018). Their current therapeutic indications are listed in Table 5.

## 5. Therapies Targeting Cancer Hallmarks

**Figure 2:**
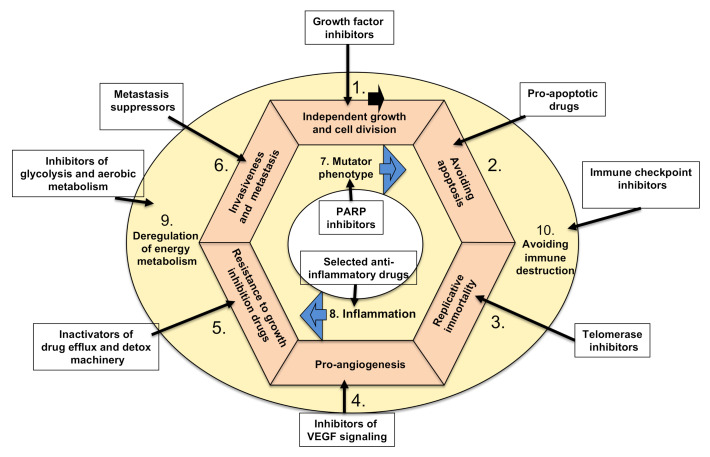
Anticancer therapies targeting cancer hallmarks.

Targeted therapies can be categorized according to their corresponding effects on one or several tumor hallmarks, as shown in Figure 2. Cancer depends on overactive proteins or signaling pathways for cell survival and growth.

These are usually changed by gene mutation or epigenetic influence. The efficacy of these drugs not only confirms particular hallmarks of tumors but also validates their therapeutic potential. Important here is the example of important current hallmark-targeted therapies. Discussing all 10 hallmarks of cancer as targets for cancer therapies is not the scope of this review, as it cannot cover the aspects of each hallmark in depth. Such an endeavor would be more suitable for a book. Nevertheless, I have chosen to cover the important elements from each hallmark, as we need to learn about them in order to create novel combinations with immunotherapies targeting the 10th hallmark, with the aim of aiding in the design of “the most suitable” strategy for each particular cancer type.

### 5.1. Targeting Tumors’ Growth Independence

#### 5.1.1. Inhibiting Cancer Proliferation

This is the most significant cancer trait. In normal tissues, growth and cell division are tightly regulated. This involves the production and control of growth factors, their receptors and signaling pathways that lead to the cell division cycle. Homeostasis is defined as the status quo of each tissue, maintained by a balance between cell death, reparation, renovation and proliferation [116,117]. The growth factor signals that control the numbers and positions of cells within tissues are probably transmitted locally. It is unfeasible, due to the experimentally difficult setup, to investigate their exact sources and targets and how they are regulated. We assume that these occur in a three-dimensional and time-based manner between a particular cell and its neighbors, and their role is to guard and conserve a particular tissue’s architecture and function.

Well known processes include growth factors binding to their cell surface (or intracellular) receptors, some of which contain tyrosine kinase domains. Some other receptors attract additional intracellular proteins possessing tyrosine, serin or threonine phosphorylation regions. All these can transduce the signal initiated by the growth-factor-binding event to the nucleus, thereby setting in motion cellular response elements in preparation for growth and division. The latter usually include changes in the survival and energy metabolism of the affected cell or tissue [117].

Although the mentioned processes are inadequately understood in normal cells, cancer cell growth and division has been better described. Cancer cells can sustain proliferative signaling by their own production of growth factors and subsequent autocrine stimulation via ligand-specific receptors or, alternatively, paracrine growth boost of the surrounding normal tissue. In addition, receptors for growth factors can be changed or deregulated such that they might be hypersensitive or active without a ligand (growth-factor independence) (reviewed in [3,15]).

Examples of the latter are signaling molecules like proto-oncogenes or “oncogenes” RAS, MYC and RAF, whose constant activation (by mutation or epigenetic alteration) defines them as oncogenes or “activated oncogenes”, respectively. Furthermore, there are three RAS genes in humans: HRAS, KRAS and NRAS. Ras proteins are small GTPases that function as transient signal transducers associated with the cell membrane. The Ras superfamily comprises the largest group (154 members out of around 220) of enzymes that exhibit picomolar affinity binding for guanosine diphosphate (GDP) and guanosine triphosphate (GTP), hydrolyzing the bound GTP to GDP and releasing orthophosphate.

Mutations of Ras, like those in codon 12, 13 and 61, convert these genes into oncogenes. The proteins change from transient status to being constantly bound to GTP and active. Permanently activated Ras mutations are among the most common in humans and account for up to 30% of all human tumors and up to 75% in particular types such as pancreatic cancer [118]. KRAS is the most frequently mutated oncogene in cancer, due to its presence in the predominant types like lung (NSCLC) [119,120], pancreatic [121] and colorectal [122] adenocarcinomas. The Welcome Sanger Institute (UK) keeps a comprehensive database involving the occurrence of RAS mutations in different human tumors.

There has been an intensive search for anti-RAS inhibitors for cancer therapy [118,123,124,125]. Molecules that inhibit activated RAS oncogenes could be grouped into direct and indirect ones. The search for the therapeutic validity of the direct competitive inhibitors, like small molecular antagonists, was largely unsuccessful until recently [126]. Probably due to the high affinity of ligand binding, it had taken five times longer time to identify an effective targeted drug for Ras (since its discovery in 1983) than for BRAF (identified in 2002). In 2019, Amgen and Mirati Therapeutics announced covalent irreversible inhibitors as a treatment for cancer that targeted one form (G12C) [126] of mutated KRAS (AMG 510 and MRTX 849) [126].

On the other hand, an indirect approach yielded results sooner. In 1989, it was discovered that the inner membrane association of the Ras protein is dependent on farnesylation, among other post-translational modifications. Hence, targeting farnesyltransferase (FTase) or using antagonists containing farnesyl-moiety were investigated as potential cancer therapeutics. As an example, we should mention salirasib, which inhibits H-, K- and N-Ras associations with the membrane. Ras further transduces a signal to the Raf-MEK-ERK MAPK pathway. Targeting the Raf-MEK-ERK pathway [123] yielded small-molecule inhibitors that include vemurafenib, a multi-kinase inhibitor, sorafenib, which is used in clinical practice for various indications (Table 1) and selumetinib (MEK inhibitor). The other branch of Ras signaling involves PI3K, AKT and mTOR. This pathway is one of the most frequently altered signal transduction pathways in human cancers [124]. Inhibitors of the PI3K-Akt-mTOR pathway are being investigated and there are several already used in clinics (everolimus, sirolimus/rapamycin and temsirolimus, as inhibitors of mTOR). A complete list of small-molecule inhibitors that are considered for anticancer therapy can be found on the web [1].

#### 5.1.2. Targeting Tumor Growth Suppressors

Tumor suppressor genes like retinoblastoma (RB1) [127] and the guardian of the genome p53 [128,129] need mutations in both hereditary copies in order to become oncogenic and promote cancer development. The strategy for cancer therapy could consist of enabling normal function of these alleles in tumors with nonfunctional retinoblastoma (Rb) or p53 proteins. This could be achieved through gene therapy, which could be ineffective, in theory, as a long-term cancer treatment, because the “rescued” Rb sufficient cancer stem cell would be eventually outcompeted by its own progeny—for example, cancer stem cells that would eliminate the gene-therapy-inserted normal functional RB1 allele. Therefore, the genetic instability characteristic of cancer would inactivate the inserted RB1 allele. While this might not be a viable option for the treatment of cancers that have both their copies of the RB1 gene inactivated, interestingly, pharmacological reactivation of another tumor suppressor p53 gene—*Gendicine*—was approved as a cancer gene therapy in China in 2003 for head and neck squamous cell carcinoma (see Section 5.2).

On the other hand, if Rb is not inactivated during carcinogenesis, molecules that could function as tumor suppressors, thus mimicking the action of Rb, would be a feasible cancer treatment. Rb is a multifunctional protein that can interact with more than one hundred cellular proteins [130]. However, its most important one is the binding and repression of a transcription factor belonging to the E2F family, thereby preventing the cell’s entry into the S phase of the cell cycle, which is characterized by DNA synthesis in preparation for replication and cell division [131]. RB1 keeps the cell in the G1 phase by ensuring that the E2F target (dimerization partner, DP) remains inactivated [130]. Furthermore, RB1/E2F-DP complex attracts histone deacetylase (HDAC) to the chromatin, which also suppresses DNA synthesis. Further, Rb functions in DNA repair and contributes to genome stability [132]. Chemical toxins that cause DNA damage in a cell can induce Rb activation. Attempts to damage the DNA of proliferating cancer cells with cisplatin or irradiation could induce Rb and prevent replication.

Most recent attempts in cancer treatment are RB regulatory factors aiming at Rb reactivation (in cancers with still functional allelic copies of RB1) such as cyclin-dependent kinase (CDK) 4 and 6 inhibitors. The FDA approved three small-molecule CDK4/6 inhibitors—palbociclib (Ibrance, Pfizer, 2015), ribociclib (Kisqali, Novartis, 2017) and abemaciclib (Verzenio, Eli Lilly, 2017)—together with aromatase inhibitors (i.e., letrozole) to treat specific breast cancer types (Table 4).

### 5.2. Inhibiting Tumor Ability to Resist Programmed Cell Death (Apoptosis)

The ability of cancer to evade apoptosis has an important role in tumor progression and resistance to therapy. Leukemic cells from patients with chronic lymphatic leukemia (CLL) and acute myeloid leukemia (AML) showed increased expression of B-cell lymphoma-2 (Bcl-2) protein, thereby avoiding apoptosis [133,134,135]. Experiments with selected tumor cell lines showed that by increasing the levels of intracellular Bcl-2-associated-X protein (Bax), these characteristics could be reversed. Bax can release proapoptotic factors from mitochondria, like cytochrome C, and can sensitize breast cancer cells to anticancer therapies. The drug that has anti-proliferative effect and can induce apoptosis (in breast cancer cells in vitro) is a naturally occurring antioxidant, 3,5-dimethoxy-4-hydroxystilbene (pterostilbene) [136]. A novel compound, venetoclax blocks the anti-apoptotic Bcl-2 protein, leading to programmed cell death [110]. In combination with azacitidine in previously untreated patients with AML, it showed a promising result, and it will hopefully be effective in some solid cancer types in trials in the future. Namely, overall survival was longer and the incidence of remission was higher compared to those who received azacitidine alone [137].

In addition to stimulating the expression of G1/S transition genes, E2F can induce the expression of pro-apoptotic genes. However, many cancers have developed signaling pathways that can counteract E2F and thus avoid death. These cancer-protective novel pathways are targets for new treatments.

The most significant protein involved in apoptosis is p53, which also functions as a tumor suppressor [138]. It is the most frequently mutated gene found in over 50% of human cancers. It was shown that the loss of p53 most often results in an aneuploidy phenotype [139].

The p53 gene encodes proteins that bind to DNA and regulate other genes’ expression, acting as a cellular guardian in order to prevent mutations of the genome [138,139]. P53 can activate DNA repair and stop the cell cycle at the G1/S checkpoint during the reparation, and it can initiate apoptosis, if DNA damage proves to be overwhelming and irreparable. Thus, p53-mediated cell death presents an opportunity to research molecules that could activate programmed cell death and be candidates for novel cancer therapies. However, there are difficulties along this pathway of drug discovery. Recent findings show that different isoforms of p53 have different cellular mechanisms for prevention against cancer [128]. The isoforms of p53 are differently expressed and distributed in various tissues. This can cause tissue-specific cancers, each with variable molecular mechanisms of anti-apoptotic abilities. The study of the dynamics of p53, together with its antagonist Mdm2 (or Hdm2 in humans), points to their concentration oscillation, which increases if exposed to oncogenic or DNA-damaging factors [140]. One isoform (a splice variant) of p53 even enhances the stemness of breast cancer cells and thus switches from tumor suppressor into an active oncogene form [141]. Thus, the models that use this current understanding of p53 dynamics could be used for novel pharmacological drug discovery. Restoration of endogenous p53 function could induce apoptosis and reduce the cell growth of, for example, lymphomas to normal levels in experimental animals. A gene therapy, *Gendicine* (China, 2003), uses adenovirus as vehicle to transform (head and neck carcinoma) cancer cells with a functional copy of the p53 gene in order to render them less proliferative.

### 5.3. Targeting Tumors’ Ability to Divide Indefinitely by Prolonging Telomeres

Telomeres are ends of chromosomes that consist of repetitive TTAGGG DNA elements, which are added by a specific enzyme, telomerase. Telomerase is a ribonucleoprotein complex that maintains telomere length in human stem cells and in around 85% of cancer cells [142]. There is an alternative telomere lengthening process that is used in 15% of cancers [143]. Shortening telomeres, which regularly comes with each cell division in normal somatic cells, leads to growth arrest, known as cellular senescence. Thus, the long-term stability of telomere length provides cancer cells with unlimited proliferation potential, and due to telomerase overexpression, cancer can grow indefinitely [2]. In detail, this process proceeds through a phase when RB-deficient and p53-deficient cells can continue to undergo telomere shortening, which leads to “telomere crisis” in many cancers. Telomere crisis can cause a multitude of genomic aberrations. Telomerase activation allows tumor cells to escape from crisis but at the cost of damaged genome integrity (reviewed in [144,145]). Inhibiting telomerase is a viable option for anticancer drugs as therapeutics. Current drugs that are designed to directly inhibit telomerase include its antagonist GRN163L (imetelstat, Geron), a telomerase template inhibitor, which has been under scrutiny for refractory myelofibrosis since 2019 (FDA) and has the potential to be a successful treatment in terms of a reduction in cell tumorigenicity and invasiveness [146]. Telomerase therapeutic vaccines (with over 30 different peptides currently in clinical trials) also offer the potential to stimulate the killing of cancer cells by increasing the activity of telomerase-specific cytotoxic (CD8) and helper (CD4) T cells (reviewed in [147,148]). Unfortunately, so far, the first vaccine with peptides derived from human telomerase did not result in favorable clinical outcomes in advanced pancreatic adenocarcinoma patients [149,150].

There are many other drugs in development for cancer therapy that can be used even in combination, including direct (MST-312) and indirect (tankyrase inhibitors) telomerase inhibitors [151,152]. The guanine-rich oligonucleotides (GROs) are able to stabilize telomeres (and thence prevent the action of telomerase) and consequently decrease cancer cell immortality [153].

Nevertheless, therapeutic drugs that promote telomere erosion via direct or indirect telomerase inhibition have shown limited improvement in cancer patient prognosis. Despite losing immortality, cancers are still viable and unaffected by the loss of telomerase activity. Additionally, some cancers can develop resistance using alternative lengthening of telomeres [154]. It is possible that there are some additional functions related to the telomere complex. Moreover, the effects of telomerase inhibition on stem cells are still not fully understood and require further study.

Therefore, a combinatorial therapy with forms of anticancer treatment targeting other hallmarks is desirable in the future testing of novel therapeutics that inhibit telomerase. These would eventually have to be assessed with regard to the influence of telomerase inhibition on life expectancy and health and should be evaluated for their benefit–risk balance [155,156].

### 5.4. Targeting Tumors’ Ability to Induce Angiogenesis

Among the first drugs that were tested in this area were inhibitors of cancer processes that include invasion with concomitant angiogenesis, as well as metastasis. These processes include enzymes that have a role in tissue homeostasis, such as the matrix metalloproteinases (MMPs), which are a family of zinc-dependent proteinases that are involved in the degradation of the extracellular matrix. MMPs play an important role in physiologic processes like wound healing. However, they can also facilitate tumor growth, invasion, angiogenesis and metastases [157,158]. Overexpression of MMPs by tumors has been associated with worse prognosis in some cancer types [159,160]. Pre-clinical data have demonstrated that interfering with MMP expression inhibits the metastatic and tumor growth, including in a breast cancer model [161]. This has led to the development of a number of MMP inhibitors, such as batimastat and marimastat, which are low-molecular-weight peptidomimetic inhibitors [162]. Unfortunately, these early preclinical studies were not repeated in clinical trials, as a phase 3 trial with marimastat showed no effect in prolonging progression-free survival when used after first-line chemotherapy for metastatic breast cancer [163]. Thus, marimastat’s development was halted after 2004.

Vascular endothelial growth factors (VEGF) represent a group of cytokines (A–E) exhibiting the ability to increase the growth of new blood (or lymph) vessels in embryonic tissues via tyrosine kinase VEGF receptors 1–3. Each tissue growth larger than 2–3 mm in diameter needs the support of oxygen to function. Thus, hypoxic tumor/cancer-growing tissue stimulates the ingrowth of blood vessels via VEGF [164].

Drugs such as aflibercept, bevacizumab, ranibizumab and pegaptanib can inhibit VEGF action and thus control the development of blood vessels. A monoclonal antibody, bevacizumab (Avastin, Roche) is an example of an angiogenesis inhibitor that works by suppressing the action of VEGF-A (FDA, 2003, Table 1). Thus far, it is given for therapy of colon, lung, renal cell cancer and glioblastoma. Recently (2018), the FDA approved bevacizumab in combination with chemotherapy (carboplatin and paclitaxel) for stage III or IV of ovarian cancer after surgical removal of the tumor. Interestingly, in 2011, the same agency withdrew its 2008 approval for metastatic breast cancer indication, claiming that there was no evidence for life extension or improved quality of patients’ lives. However, it remains approved for breast cancer in other countries, including Australia. The drug has been classified as a “specialty drug” that may have unusual side effects or may be unusually expensive. Furthermore, a biosimilar of bevacizumab called Mvasi (Amgen) was approved for several cancer therapies in 2017.

The mesenchymal-to-epithelial transition (MET) tyrosine kinase receptor and its ligand, hepatocyte growth factor (HGF), are overexpressed and/or activated in a wide variety of human malignancies. HGF is a potential target for anticancer therapy due to its angiogenic properties. Inhibitors of cMET-HGF interaction include foretinib (GlaxoSmithKline), which targets cMET and VEGFR-2 kinase enzymes, which are being assessed for cancer treatment. Despite its development to treat a number of cancer indications being halted in 2015, recently, foretinib was shown to be able to inhibit cancer cell stemness and diminish the proliferative ability in some gastric cancers by counteracting CD44 and cMet tyrosine kinases [165]. Anlotinib is a novel small-molecule multi-target tyrosine kinase inhibitor that can inhibit tumor angiogenesis and tumor cell growth. It is currently being investigated in a clinical study on the treatment of recurrent glioblastoma (which includes patients with mutations in VEGFR, Kit, PDGFR and FGFR genes) [166].

### 5.5. Targeting Growth Suppressor (Drug) Resistance of Cancer Cells

Chemotherapeutic drug resistance in cancer cells depends on five groups of mechanisms: 1. Increased DNA repair ability, 2. Enhanced efflux of drugs, 3. Elevated metabolism (catabolism) of drugs, 4. Genetic and epigenetic factors, and 5. Growth factor compensation (reviewed in [167,168,169,170]). Counteracting these represents potential treatment opportunities for cancers that develop drug resistance. Here are the potential candidates:

1. DNA repair endonucleases (xeroderma pigmentosum group F, XPF and excision repair cross-complementing protein group 1, ERCC1) involved in the nucleotide excision repair (NER) pathway, are central for the effective repair of DNA damage induced by platinum-based agents [171]. Cancer can become resistant to, for example, cisplatin by overexpressing both XPF and ERCC1 proteins [172]. Compounds like E-X PPI2 and E-X AS7 were identified as XPF-ERCC1 pathway inhibitors [173] and are a viable option for treating such cancers. However, Pt-DNA lesions depend, besides the NER pathway, also on the homologous recombination (HR) pathway. Moreover, there are mismatch repair (MMR) [174] and interstrand crosslink (ICL) repair [175] mechanisms which might be involved too. Thus, together with the latter two, Replication protein A (RPA) inhibitors [176], ataxia teleangiectasia-related (ATR) kinase inhibitors [177], DNA-PKcs inhibitors [178], HR inhibitors [179] and translesion synthesis (TLS) inhibitors [180,181] are all potential drugs for restraining multidrug resistance (MDR) in cancer that depends on overactive DNA repair machinery.
2. Enhanced efflux of drugs is exemplified by the P-glycoprotein (P-gp) family that binds ATP (ATP-binding cassette [ABC] transporters) [182]. Overexpression of P-gp, multidrug-resistance-associated protein and breast cancer resistance protein (BCRP) that are present in the cell membrane are responsible for a major portion of multidrug resistance in cancer [183,184,185]. This is because they regulate the absorption, excretion and distribution of many drugs used as chemotherapy. They decrease the toxic high intracellular concentration of the administered chemotherapeutic drug, diminishing its bioavailability and therapeutic effect. P-gp, as a multidrug membrane transporter, normally pumps chloride out of the cells and can bind to a variety of chemotherapy agents, including doxorubicin, vinblastine and taxol [183]. Finding a remedy for MDR has been a long-standing challenge in cancer therapy. There is a number of P-gp inhibitors that show significant anticancer effects in various experimental studies, but unfortunately, none have entered clinical trials except sitravatinib [186], which is a receptor tyrosine kinase inhibitor that could reverse multidrug resistance in vitro. Sitravatinib is an orally available, potent small-molecule inhibitor of a closely related spectrum of receptor tyrosine kinases (RTKs) including MET, Axl, MERTK, VEGFR family, PDGFR family, KIT, FLT3, Trk family, RET, DDR2 and selected Eph family members. Sitravatinib is currently in clinical trials with nivolumab (which targets hallmark #10) for metastatic lung cancer (NSCLC, phase 2) [186] and also for renal cell carcinoma (phase 3) [187].
3. Elevated metabolism of xenobiotics. Isoforms of cytochrome (CYP) are important for the drug degradation and detoxification of xenobiotic [188] and endogenous compounds like estrogen and testosterone [189,190]. Overexpression of CYP1B1 has been observed in various cancer types, especially those which are estrogen responsive, and is linked with enhanced resistance to a variety of drug therapies, including docetaxel [191]. Glutathione (GSH) maintains cellular redox homeostasis. Cancer cells show higher reactive oxygen species production than normal cells. Due to the increased requirement for energy for proliferation, cancer produces an antioxidant defense system to balance out the elevated oxidant state. Many cancer types show overexpression of GSH. Furthermore, GSH detoxifies extracellular toxins, drugs and biotics and can increase cancer multidrug resistance [192,193]. The inhibition of the GSH antioxidant defense system (flavonoids and chalcone derivatives) [194] could prevent the resistance of cancer to the used chemotherapeutics and is a reasonable strategy to avoid multidrug resistance of cancer.
4. Genetic mutations in genes involved in MDR have been common reasons for cancers resisting selected drug therapy. Amplifications of genes that are being repressed by the chemotherapy—for example, dihydrofolate reductase (DHFR) in methotrexate chemotherapy—have been seen in many cancers, including leukemias. Recently, epigenetic modifications were found that can also increase MDR in cancers. There are various miRNAs that influence the sensitivity of cancer cells against anticancer agents which target genes related to cell proliferation, cell cycle and apoptosis (reviewed in [169]). Furthermore, histone deacetylase inhibitors (like mocetinostat) or histone kinase inhibitors (i.e., CUDC-101, -907) that alter the expression of various genes whose products are involved in diverse mechanisms of chemoresistance of cancer could be useful for anticancer resistance prevention, perhaps best tested in combinatorial treatments. Likewise, mocetinostat has been undergoing clinical trials since 2016 to evaluate the clinical activity in NSCLC (phase 2, ends in 2021) and RCC (phase 3, ending in 2024), in combination with three separate investigational agents, one being the immune checkpoint inhibitor nivolumab, and comparing its efficacy against sitravatinib (a multi-targeted RTK inhibitor) or glesatinib (also a multi-targeted tyrosine kinase inhibitor) [186,187].
5. As additional manifestation of chemoresistance, cancers can upregulate the autocrine production of various cytokines including growth factors such as IL-1, Il-4, Il-6 and IL-8. Biologics like IL-6 (siltuximab, Sylvant) or IL-6R inhibitors (tocilizumab, Actemra; sarilumab, Kevzara) have been suggested as therapy to counteract cancer MDR, both of which are being tested for the treatment or prevention of SARS-CoV-2 severe pneumonia [195]. Furthermore, FGFs present in solid tumors not derived from cancer cells but from surrounding accompanying cells in a tumor mass can provide resistance to chemotherapy, such as 5-FU, DOX and paclitaxel, which have different mechanisms of action. Suramin, an inhibitor of FGFs, could reverse this resistance in vitro. Small-molecule inhibitors of FGFs are currently in trial for the treatment of glioblastoma [166].

In the future, tumor therapies that target cancer MDR might include different types of drugs in combination therapies corresponding to various aspects of MDR mechanisms, listed above [169].

### 5.6. Targeting Cancers’ Invasive Spreading with Metastasis

As cancer progresses, it becomes more aggressive by beginning to breach the surrounding tissue structure. This invasion of the microenvironment depends on the deregulation of contacts with other cells and the detachment of some tumor cells from the solid tumor mass. Metastasis, on the other hand, is a complex sequential process that involves multiple cells, factors and stages, leading to the dissemination of cancer to other tissues and organs. It can disseminate via lymph and blood. Brain, bone, lung and liver were found to be the most frequent sites of hematogenous spread from some solid tumors (reviewed in [196,197]).

Remodeling the extracellular matrix (ECM) is the major factor in solid cancer progression, invasion and metastasis. Metastasis initiation genes determine dissociation, detachment and invasion of cancer, and include promoters of cell motility, epithelial mesenchymal transition (EMT), and ECM degradation. In addition, they contain genes that modulate angiogenesis and evasion of the immune cells’ attack. EMT is a spectrum of transitional stages shifting through invasive, metastatic and differentiated phases during either single-cell dissemination or collective migration (reviewed in [197]). Since EMT might be required for metastasis initiation, drugs inhibiting cell motility and EMT can be important cancer metastasis therapeutics.

The natural dietary compound pterostilbene, found in blueberries, in addition to its antiproliferative and proapoptotic abilities on some cancer cell lines [136], has also antitumorigenic potential on cancer stem cells and anti-metastatic activity in vitro [198].

Dietary restriction of asparagine or its decrease through L-asparaginase treatment can diminish metastatic spread in experimental settings [199].

Metastasis suppressor genes (reviewed in [200]) target the MAPK pathway (i.e., NMO9, RKIP, MKK4,6,7), adhesion proteins (KAI1, E-cadherin, CD44), cytoskeletal signaling (Gelsolin, Rho-Rac pathway, DLC-1), G-protein coupled receptors (KISS1, PKA/C pathway, AKAP12) and apoptotic pathway (Caspase-8) and are found mutated in various cancers that have metastasized. Epigenetic changes can also play a role in metastasis [201].

Significant amounts of exosomes are released by primary tumor cells. These can transfer invasion-promoting factors, including various miRNAs, to tumorigenic cancer cells [202]. Counteracting all these processes might diminish the metastatic spread of cancer and be potential novel treatments.

Despite these advances, the metastasis process remains poorly understood and thus a pharmacological approach to inhibiting metastasis is still limited. There has been a resurgence of interest in new antitubulin reagents that could reduce metastasis, such as vinca alkaloids or taxans (Table 2) with novel properties like higher cancer specificity, fewer side effects and insensitivity to chemoresistance.

### 5.7. Targeting Tumors’ Mutator Phenotype by Counteracting Their Ability to Increase Genetic/Epigenetic Instability

While normal cells have a low rate of spontaneous mutations [203], cancer cells display widespread DNA and chromosomal changes [204,205]. Such increased genome instability selects more malignant forms of tumor cells and speeds up the evolution of invasive and metastatic forms of cancer [5]. The key elements and genes playing a part in genome maintenance and replication are conserved throughout evolution, with a few exceptions (reviewed in [206]). The orthologs of genome integrity network genes in single-cell organisms were discovered to be dysregulated in cancer. These include cohesion mutations [207] and the microsatellite instability linked to the mismatch repair pathway [208]. The mismatch repair pathway is a conserved surveillance system that identifies and exchanges wrongly incorporated bases in DNA [209]. Mice lacking functional mismatch repair proteins have dysregulated genome integrity and are predisposed to the occurrence of spontaneous cancers [210].

In bacteria, the mutator phenotype helps in adaptation and survival, and it could be reversed (or selected against) once adaptive mutations are fixed [211,212]. In contrast, in cancer, we assume that the mutator phenotype is deleterious as it can initially select for cancer cells that can counteract environmental suppression by immunity, tissue homeostasis forces or drugs used to inhibit growth. The mutator phenotype thus enhances the speed of cancer evolution in a patient. It is thought that cancer stem cells could change their genome instability to generate heterogeneity of cells, which could be selected, or/and stabilize survivors that have endured immune attacks. Therefore, to counteract the genome instability of cancer stem cells, it is important to find strategies that prevent mutator phenotypes of cancer (reviewed in [206]).

Furthermore, recent advances show that innate immunity is important in sensing the presence of cytoplasmic DNA derived from genomic instability events, such as DNA damage and defective cell cycle progression. This is achieved through the cyclic GMP-AMP synthase (cGAS)/stimulator of interferon genes (STING) pathway [213]. Therefore, inhibition of the cGAS/STING pathway can be a viable anticancer therapy [214].

### 5.8. Targeting Tumor-Promoting Inflammation by Anti-Inflammatory Drugs

Persistent production of inflammatory mediators can lead to tissue and DNA damage. In turn, this can lead to increased risk of developing cancer. In addition, mutated tumor cells are able to generate an inflammatory environment that can accelerate cancer development. Recently, cyclic GMP-AMP synthase with downstream effector, stimulator of interferon genes (STING), was shown to be essential in antimicrobial immunity as well as for antitumor immunity. The resulting cytokines produced by this pathway, especially type I interferons, can connect innate immunity with adaptive immunity against cancer. Chronic STING activation can lead to a tumor promotor phenotype and eventually to malignancy. Hence, inhibiting the pro-inflammatory effect of tumors can slow down tumor growth and cancer development (reviewed in [214]). Of all the current anti-inflammatory drugs, the most studied ones and those used in clinics are aspirin, non-steroidal anti-inflammatory drugs (NSAIDs) including diclofenac, colecoxib, sulindac, ibuprofen, piroxicam and corticosteroids (dexamethasone, hydrocortisone, prednisone). The cancer prevention effects of aspirin and NSAIDs are well known [215]. Studies also show that anti-inflammatory drugs display a range of effects on the immune system, angiogenesis and tumor metabolism [216]. Furthermore, they could reduce invasiveness, metastasis, increase apoptosis and promote sensitivity to radio- and chemotherapy [217].

### 5.9. Targeting Tumors’ Metabolic Shift from Oxidative to Anaerobic Glycolysis

Cancer cells preferentially use glycolysis to generate energy for proliferation and growth rather than oxidative phosphorylation in the mitochondria [218,219]. This shift in energy generation causes a “more expensive” pathway of creating ATP and is seen in many proliferating cancers, even in the presence of oxygen, due to the influence of some key metabolism regulator genes like c-Myc and TOR [220]. Hence, inhibiting the metabolic reprogramming (i.e., anaerobic generation of energy via c-Myc activation) could be a viable option in cancer treatment.

Glucose transporters (GLUTs), particularly GLUT1 and GLUT3, which deliver the carbohydrate substrate in both glycolysis and oxidative phosphorylation pathways, were found to be overexpressed in most cancer cells [221,222]. Due to rapid proliferation, cancer cells are ravenous for energy uptake (glucose). Hence, an inhibitor of glucose uptake, like a compound “TH-G313B”, having negligible cytotoxicity, which was effective in inhibiting GLUT-mediated transfer and constrained cancer cell growth both in vitro and in vivo, would be an ideal candidate for a novel cancer treatment [223].

Glycolysis was shown to be used in both embryonal and induced pluripotent stem cells [218]. Interestingly, cancer stem cells also use glycolysis, when deprived of oxygen, in contrast to their progeny—differentiated cancer cells [218,220]. This flexibility of cancer stem cells to efficiently gain energy from anaerobic glycolysis when oxidative phosphorylation is blocked is thought to be a specific target for therapy of cancer. The “two metabolic hit strategy” for the eradication of cancer stem cells has been proposed. The first hit would undermine oxidative phosphorylation in mitochondria of cancer cells (via, e.g., doxycycline, which effectively reduces cellular respiration, by targeting mitochondrial protein translation), and the second hit would prevent glycolysis by its inhibitors (e.g., vitamin C) [224]. This dual- rather than single-pathway inhibition strategy would aim to eradicate the remaining resistant cancer stem cells and, in theory, prevent the invasiveness, metastasis and reappearance of cancer.

### 5.10. Targeting Cancers’ Escape from Destruction by the Immune System Using Various Immunotherapies (Biological and Cell-Based)

**Figure 3:**
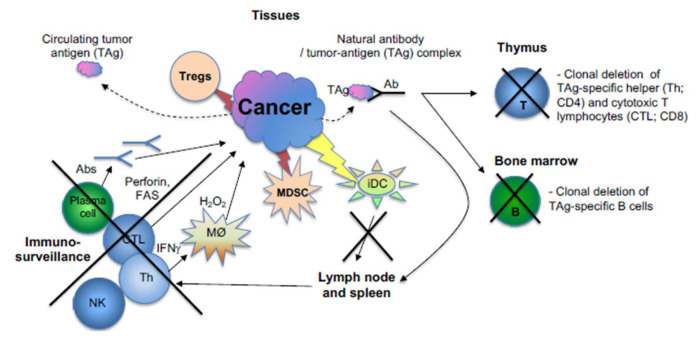
Avoiding immune cells’ attack: 10th hallmark of cancer.

The immune microenvironment around the tumor plays a major role in tumor development and possibly metastasis. The cancer immunosurveillance hypothesis has proposed that the immune system checks the integrity of an organism by innate and adaptive immune responses. The immune system contributes to homeostasis by protecting the body and tissues, overseeing the development and balancing the growth and maintenance of organs and tissues [225,226]. In Figure 3, multiple avenues of cancer avoidance of immunosurveillance are shown. They are as follows:

1. Cancer can directly (or indirectly, by secreting a putative factor) diminish the function of either activating or effector T (or B) cells by engaging at least one of the following molecules: CTLA-4, PD1, VISTA, BTLA, CD160, CD244, LAG-3 and TIM-3 [227].
2. Deletion of T cell clones (potential antitumor) in the thymus (migrating tumor cells or antigen-presenting cells which have acquired tumor antigens elsewhere) [228].
3. Deletion of B cell clones (potential antitumor) in the bone marrow (by presenting tumor antigens to developing B cells) [229].
4. Tregs: Cancer can activate or induce suppressor T cells (Tregs) that suppress antitumor T, B and NK immunosurveillance cells [230]. The development of regulatory T cells in the thymus and periphery of the immune system [226] is known, and their role was suggested to be of paramount importance for the immunosurveillance of cancer [225].
5. Cancer could inhibit the maturation of dendritic cells (DC) [231] and, as such (remaining immature, iDC), they cannot activate the adaptive immune response against the tumor [227,232].
6. Myeloid-derived suppressor cells (MDSC) are cells that can inhibit the adhesion of other leukocytes onto the endothelial surface in the tumor [233].

**Figure 4:**
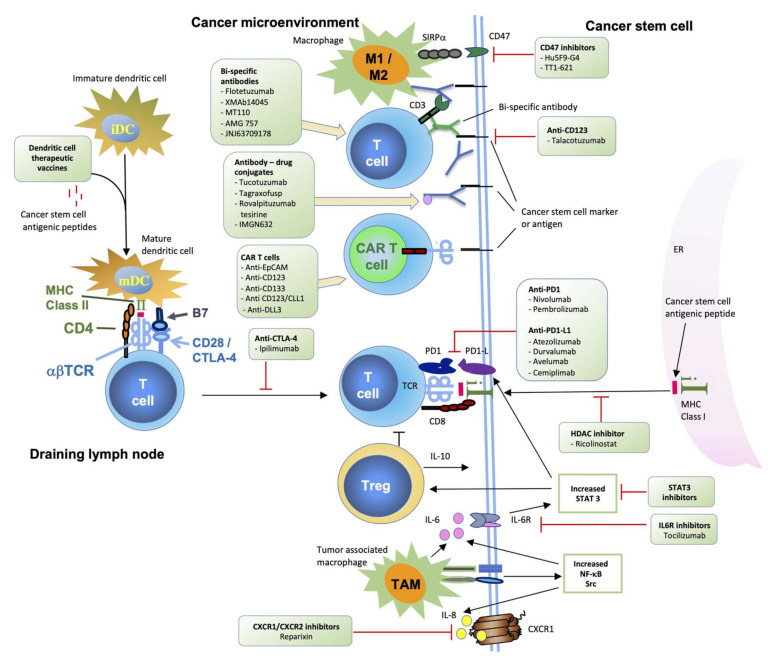
Targets of anticancer-stem-cell immunotherapies.

Immune checkpoints are defined as regulatory processes during the adaptive immune response to immunogenic proteins (antigens) that have been expressed (as modified normal antigens, called neoantigens) by cancer as a way of evading its destruction by immunocytes (Figure 3). The first checkpoint regulates the antigen presentation phase in the initiation of the response. There is a dual molecular interaction between the antigen presenting cell (APC) and the T cell causing the activation of the latter. The first is called stimulation, as it defines the specificity of interaction with respect to immunogenic tumor antigen. It is between the T-cell receptor (TCR) and the tumor antigen’s peptide imbedded within the groove (made by alpha helices) of the Major histocompatibility complex (MHC) class II molecule. The second interaction, called co-stimulation, occurs between B-7 (CD80 or CD86) and CD28 molecules (Figure 4, on the left), and is a prerequisite for the activation of T helper (CD4) cells to generate antitumor effectors (capable of destroying cancer cells, bearing a particular tumor antigen, either alone or by helping cytotoxic CD8 T cells to do so). However, there is a catch. Another B-7 binding molecule, CTLA-4, is also expressed on the T cell, and it is upregulated after activation. It outcompetes the CD28 molecule in the interaction with the B-7 molecules, because it has higher affinities for both ligands. The CTLA-4 function is co-inhibitory, and it diminishes the activation of T cells. It is thought that CTLA-4 constraints the immune response representing perhaps its negative feedback loop. It is believed that cancer uses this mechanism to escape T-cell attack. For example, cancer could enhance the expression of the CTLA-4, and thus downregulate anti-tumor T-cell responses (reviewed in [234,235]). Ipilimumab is a biologic that can inhibit this inhibition (resulting in activation of anti-tumor immunity) and consequently provide an immune attack against a number of cancers (Figure 4) [86,87]. It has also shown efficacy in clinical use [230].

Another checkpoint is in the effector phase of the immune response (Figure 4, on the right).

The programmed death 1 (PD1) molecule is expressed on effector (activated) cytotoxic T cells [236] (able to destroy tumor cells), and can interact with its ligands, either PD-L1 or PD1-L2, on target (tumor) cells. This interaction causes antitumor T cells to become inactive and unable to kill cancer cells. Thus, the expression of PD1 ligands is a means of evading immune destruction [237,238]. The function of the PD1/PD1-ligand interaction is still not fully understood. It is thought that they are needed when T cells become exhausted, and they represent a sign of senescence, when a new set of these cells should become involved. Therefore, for T cells, this interaction is an inhibition of the immune response to the particular target expressing the ligand. PD1 ligands are expressed on B lymphocytes, natural killer cells, myeloid, endothelial, epithelial and on cells within the tumor microenvironment of many types of cancer.

Pembrolizumab and nivolumab are biologics binding to PD1 that can inhibit its function (Figure 4). They (and other biologics directed against PD1 ligands) were successfully used in therapy against many (mostly) PD1 ligand positive cancers in recent years, with metastatic melanoma being the most exclusive example. In addition, boosting T cell memory (probably by targeting other than PD1 molecules on T cells) may lead to more durable anticancer responses than seen with conventional therapies [239].

The avoidance of destruction by the immune system is the cancer capability that has gained the most interest in modern cancer therapies (reviewed in [17]). There are, however, shortcomings related to immune checkpoint therapies [240].

Firstly, not all patients respond to immunotherapies. Immune checkpoint inhibitors demonstrate the best success in advanced melanoma (stage 4), with 40% response rate. Other cancers have progressively less responders [239]. Here, we anticipate that BTLA, Tim3, CD160 and LAG3 targets might increase the percentage of responders. Similarly, tumor neoantigens would also contribute, as they would recruit more clones of T cells that could attack cancer cells. While we could be optimistic with these prospects, the disadvantages of immunotherapies are their side effects and adverse events.

The main adverse event in novel cancer immunotherapies is autoimmunity of some kind. Treatments for the majority of such adverse events are symptomatic and conservative in nature, consisting of corticosteroids or their derivatives. The algorithm for treating such adverse events in novel therapies with immune checkpoint inhibitors has been designed and only rarely includes cessation of the anticancer immunotherapy [241].

Do exacerbations of autoimmunity occur at a higher rate among patients with underlying autoimmune disorders? This is not known, as patients with autoimmune disorders were excluded from clinical trials with immune checkpoint inhibitors. However, several cases have recently been reported of patients with autoimmune disorders successfully being treated with ipilimumab without exacerbation of their underlying autoimmune disorder. Clinicians should weigh the potential benefits of anti -CTLA-4 and -PD-1/PD-L1 therapy in treating life-threatening malignancy against the theoretical risk of exacerbating an underlying autoimmune disorder [240].

Immune related adverse events (irAEs) include dermatologic, gastro-intestinal, hepatic, endocrine and other less common inflammatory events. Dermatologic adverse events (AEs) are the most common irAEs. The development of rash, pruritus, alopecia and vitiligo (in 20–30% of patients) may lead to dose modifications and/or termination of therapy [241].

A meta-analysis to ascertain the incidence and risk of developing dermatologic AEs during treatment with the PD-1 inhibitors, pembrolizumab and nivolumab was performed. Skin rash, pruritus and vitiligo are the most commonly reported AEs, although they appear to be primarily low-grade and manageable. These were suggested to be used as predictive biomarkers for the toxicity of anticancer treatment [242].

Among the PD-1 inhibitor-induced skin rashes, the maculopapular morphology is most frequent, often portraying a lichenoid tissue reaction/interface dermatitis on histology [242].

In conclusion, the adverse events in novel cancer immunotherapies comprise mostly autoimmune phenomena, either organ-specific or systemic. Therapeutic countermeasures are palliative and symptomatic, including temporary immunosuppression with corticosteroids, tumor necrosis factor antagonists, mycophenolate mofetil or other agents [241]. The most intriguing result was that most, if not all, such patients entered remission regarding the autoimmunity within several months of the treatment. This is different from non-cancer patients with autoimmune diseases. Namely, the disease is never cured with the same medicines. The autoimmune disease in patients as a primary disease (that do not have cancer) may have a constant driving mechanism that cancer patients lack, which leads the immune cells (T and B) to constantly attack their own tissues.

The current challenge is to understand whether a combination therapy of an immune checkpoint inhibitor with chemotherapy as a first-line therapy would be a promising course of treatment. Novel candidates for immune checkpoint inhibitors like BTLA, Tim3 and CD160 (Figure 3) would provide additional hope that eventually cancer can be sensitized to be vulnerable to immune cells’ attack. They could be then combined with chemotherapeutics including already existing small-molecule inhibitors of hallmarks of cancer. It has been suggested to enhance antigen presentation and the maturation of DCs via Vinca-alkaloids, Cisplatin, Methotrexate, 5-FU and Anthracyclines. Drugs that could deplete Tregs and other suppressors (MDSC) could be targeted by Anthracyclines, Taxanes, Cyclophosphamide, Cisplatin, Gemcitabine and 5-FU [243]. Novel candidates inhibiting intracellular signaling cascades of cancer characteristics would perhaps also be within the scope of potential treatments in the future. Lastly, neoantigens either cancer-type specific or patient-specific, could be used for stimulating specific antitumor T cell responses in combination with immune checkpoint inhibitors, as a precise personalized immunotherapy.

## 6. Therapies That Target Epigenetic Changes in Cancer

The cancer would develop from a single cell by acquiring DNA mutations and epigenetic alterations, thereby escaping homeostasis. It is hypothesized that the initial event in carcinogenesis occurs by an epigenetic reduction in the expression of one or more DNA repair enzymes. This epigenetic alteration would lead to the accumulation of DNA damage and subsequently to the selection of other hallmarks of cancer in daughter cells. Cancer cells exhibit a changed DNA methylation pattern [244]. It has been established that epigenetic changes (including CpG-island methylation, histone modification patterns and microRNA levels) increase during tumor progression (from normal tissue over hyperplasia, metaplasia, neoplasia to invasion stages) (reviewed in [201]). Benign tumors lack mutations or epigenetic changes in invasiveness and metastasis and perhaps in several other hallmarks.

Drugs that reverse the changes made by the epigenetic modifications might be of use in cancer treatment. In the last two decades, the development of anticancer drugs has concentrated largely on inhibitors of epigenetic modifying enzymes such as the DNA methyltransferases (azacitidine, decitabine), histone acetyltransferases, histone deacetylases (ricolinostat, vorinostat, romidepsin and panobinostat) and other histone modifying enzymes (like lysine and arginine methyltransferases). The hypomethylating drugs azacitidine and decitabine are used to treat myelodysplastic syndrome in preventing the onset of leukemia [245]. The inhibitors of histone deacetylases (HDAC) “suberoylanilide hydroxamic acid” or vorinostat (Zolinza, Merck) and the product of *Chromobacterium violaceum*, romidepsin (Istodax, Celegene), are approved for the therapy of cutaneous T cell lymphoma [246,247]. Panabinostat (Farydak, Novartis) [248] is presently approved (2015) in the treatment of multiple myeloma, relapsed or refractory types in patients who have already been treated with bortezomib (Velcade, Takeda, Millennium Pharms), a proteasome inhibitor and an immunomodulatory treatment such as lenalidomide (Revlimid, Celgene) or thalidomide (Thalomid, Celgene) (Table 1). Interestingly, lenalidomide, an analogue of teratogenic thalidomide, was found to promote anti-inflammation, because it inhibits COX2, decreases the secretion of pro-inflammatory cytokines (IL-1β, TNFα, IL-6 and IL-12) and increases the secretion of anti-inflammatory cytokines (IL-10) from PBMCs. In addition, it induces T cell proliferation by stimulating the secretion of IL-2 and IFN-γ. Furthermore, it inhibits myeloma and Burkitt lymphoma cell proliferation. It inhibits angiogenesis and induces apoptosis via G1 arrest of tested in vitro cell lines [249].

HDAC inhibitors entinostat and quisinostat can disrupt the interactions between the tumor microenvironment and host immune surveillance [250]. HDAC inhibitors can increase the immunogenicity of cancer in numerous ways, including the upregulation of the expression of tumor antigens, tumor antigen presentation on antigen-presenting cells and co-stimulation molecules in cancer cells (Figure 4). Entinostat can also inhibit the function of suppressive T regulatory (Treg) cells through the acetylation of the STAT3 transcription factor (Figure 4) [17].

In mammals, over 60% of genes that are transcriptionally active are regulated by miRNAs. The epigenetic silencing of miRNA by aberrant DNA methylation in cancer is a frequent event [244]. Thus, microRNA therapies are potentially useful novel therapies against cancer, and types of anticancer drugs that modulate miRNAs’ functions span from oligonucleotides complementary to specific miRNAs to enzymes involved in their expression and activity.

The nutritional signals that can modulate the activity of epigenetic enzymes have been under scrutiny, and this area of cancer research and treatment has certainly been insufficiently explored.

## 7. Concluding Remarks

Many of the cancer hallmarks are regulated by partially overlapping intracellular signaling pathways. Thus, in response to a targeted treatment, cancer stem cells may reduce their dependence on a particular hallmark capability, becoming more dependent on another. Therefore, cancer therapies targeting a single hallmark could induce an adaptive change in dependence on other capability traits, which, in turn, would constrain the efficacy of hallmark-targeting therapies. This could perhaps explain the shift in cancer cells towards more invasive and metastatic forms in patients with antiangiogenic therapies [251].

This shift might allow some cancer stem cells to survive and become resistant to the applied therapy and, consequently, clinically relapse. However, since the number of parallel signaling pathways necessary to define a particular hallmark is not infinite, it should be possible to design a combination therapy to target all of these adverse effects (induced parallel pathways) of initial treatment and thus avoid, in theory so far, adaptive resistance, which is different from multidrug resistance. The strategy for incorporating these concepts into drug design for novel treatments has been previously explained [2,5,19,169].

## Figures and Tables

**Table 1:** Historical perspective of anticancer drugs: Part 1. 1949–2006.

Year of Approval	Drug (Therapy)	Category	Mode of Action	Targeted Hallmark	First Indications (Current)		
					Solid Tumors	Blood Borne	Institution or Country
1949	Nitrogen mustard (Mustine, mechlorethamine)	Chemotherapeutic	Nonspecific DNA alkylating agent; binds and crosslink DNA, prevents cell duplication	1,2	Bronchogenic carcinoma	Hodgkin’s disease, lymphosarcoma, chronic myelocytic leukemia [CML], polycythemia vera	USA (FDA)
1953	Methotrexate; 6-Mercaptopurine	Chemotherapeutic	Blocks cell cycle in S phase	1,2	Breast, ovarian, bladder, head and neck cancer, osteosarcoma, choricarcinoma	Acute lymphoblastic leukemia [ALL]	FDA
1959	Cyclophosphamide	Chemotherapeutic	Nitrogen mustard, DNA alkylating agent; crosslinks DNA, blocks cell cycle	1,2		Multiple myeloma	--′′--
1961	Vinblastine	Chemotherapeutic	Blocks cell cycle in M phase	1,2	Cancer		--′′--
1962	5-Fluorouracil (5-FU)	Chemotherapeutic	Blocks cell cycle in S phase	1,2	Cancer		--′′--
1964	Melphalan	Chemotherapeutic	Nitrogen mustard, DNA alkylating agent; crosslinks DNA, blocks cell cycle	1,2	(Childhood neuroblastoma, ovarian cancer, and mammary adenocarcinoma)	Multiple myeloma (Hodgkin lymphoma, non-Hodgkin lymphoma, ALL and AML)	FDA (EMA in 2020)
1974	Doxorubicin	Chemotherapeutic	Inhibiting eukaryotic cell growth (anthracydine)	1,2	Breast cancer, bladder cancer, Kaposi’s sarcoma	Lymphoma, and ALL	FDA
1975	Dacarbazine	Chemotherapeutic	Nitrogen mustard, DNA alkylating agent; crosslinks DNA, blocks cell cycle	1	Melanoma (sarcoma; MAID * regimen)	Hodgkin lymphoma [a part of ABVD′′ regimen]	--′′--
1977	Carmustine	Chemotherapeutic	Blocks cell cycle; nitrosourea, alkylates DNA, action not fully understood	1,2	Palliative in glioblastoma, and brain tumors	Multiple myeloma [palliative in refractory Hodgkin or non-Hodgkin tumors]	--′′--
--′′--	Tamoxifen	Chemotherapeutic	Inhibiting growth (anti-estrogen synthesis), cell cycle in G1 phase	1	Breast cancer		--′′--
1987	Ifosfamid	Chemotherapeutic	Nitro mustard, DNA alkylating agent; crosslinking DNA alkylating agent; crosslinking DNA and blocking cell cycle	1	(Testicular, ovarian, bladder, cervical, small cell lung cancer, E wing and soft tissue sarcoma, osteosarcoma, thymoma)	Hodgkin, and non-Hodgkin lymphoma	--′′--
1989	Carboplatin	Chemotherapeutic	Blocks cell cycle	1,2	cancer		--′′--
1991	Paclitaxel	Chemotherapeutic	First oftaxans, antimicrotubule agent, blocks cell cycle in M phase	1,2	Advanced ovarian carcinoma (breast, NSCLC, SCLC, opancreatic, es)		--′′--
1995	Anastrozole	Chemotherapeutic	Aromatase inhibitor (inhibits estrogen synthesis)	1	Advanced breast cancer [postmenopausal; if progressed on tamoxifen therapy]		UK, --′′--
--′′--	Tretinoin	Chemotherapeutic	Vitamin A related	1		Acute promyelocytic leukemia	FDA
1996	Oxaliplatin	Chemotherapeutic	Blocks cell cycle	1,2	Advanced colorectal carcinoma [for the treatment of 5-FU pretreated patients]		France (FDA in 2004)
1996	Topotecan; Irinotecan	Chemotherapeutic	DNA-modifying enzyme inhibitors; topoisomerase-1 inhibitors, block cell cycle in S phase	1,2	Metastastatic ovarian and colorectal carcinoma (cervical SCLC, pancreatic)		FDA
1996	Letrozole	Chemotherapeutic	Aromatase inhibitor (inhibits estrogen synthesis)	1	Early stage breast cancer [poswtmenopausal]		France (FDA in 2004)
1997	Rituximab	Biologic	Inbihiting proliferation (anti-CD20)	1		Non-Hodgkin lymphoma	FDA
1998	Trastuzumab (herceptin)	Biologic	Inbihiting proliferation (anti-EGFR2)	1	Metast brest cancer		--′′--
2001	Alemtuzumab (Campath1)	Biologic	(anti-CD52)	1		Chronic lymphocytic leukemia [CLL]	--′′--
--′′--	Imatinib (Gleevec)	Chemotherapeutic/small molecule inhibitor	Inhibit of Bcr-Abl tyrosine kinase	2	Gastro intestinal stromal tumor [GIST]	CML, ALL-Philadelphia chromosome positive	--′′--
2003	Bortezomib (Velcade)	Chemotherapeutic	Reversible proteasome inhibitor; cell growth arrest, apoptosis	2	Relapsed or refractory multiple melanoma		--′′--
2003-2	Ibritumomab tiuxetan (Zevalin)	Radionuclide-linked biologic	(Anti-CD20)	1		Non-Hodgkin lymphoma	--′′--
--′′--	Tositumomab (Bexxar)	Radionuclide-linked biologic	(Anti-CD19)	1		Non-Hodgkin lymphoma [withdrawn in 2014-15]	FDA-EMEA
2004	Cetuximab (Erbitux)	Biologic	Inhibiting proliferation (anti-EGFR signaling), inducing apoptosis	1,2	Metastatic colorectal carcinoma		FDA
--′′--	Bevacizumab (Avastin)	Biologic	Inhibiting angiogenesis (anti-VEGF)	4	Metastatic colorectal carcinoma (NSCLC, glioblastoma, renal cell carcinoma, breast, ovarian cancer)		--′′--
2005	sorafenib	Chemotherapeutic	Multi-kinase inhibitor of Ras (Raf-MEK-ERK) pathway, anti-angiogenic (anti-VEGFR2,3)	1,4	Advanced renal cell carcinomas (from 2007, hepatocellular carcinoma)		--′′--
--′′--	Exemestane (and anastrozole)	Chemotherapeutic	Aromatase inhibitor (inhibits estrogen synthesis)	1	Early breast cancer [hormone receptor positive]		--′′--
2006	Gardasil	Prophylactic Vaccine	Anti-HPV types 6,11,16 and 18	10	Prevention of cervical carcinoma		--′′--
--′′--	Thalidomide (Thalomid)	Chemotherapeutic	An immune omodulatory drug with spectrum of activities notfully charaterized	1,8		Relapsed or refractory multiple myeloma [with dexamethasone in combination]	--′′--
--′′--	Lemalidomide (Revlimid)	Chemotherapeutic (Thalidomude analogue)	An immunomodulatory drug; inhibits COX2, inhibits angiogenesis, induces apoptosis via G1 arrest	1,2,4,8		Relapsed or refractory multiple myeloma [with dexamethasone in combination]	--′′--
--′′--	Panitumumab (Vectibix)	Biologic	EGF receptor inhibitor, inhibiting proliferation and inducing apoptosis	1,2	Metastatic colorectal cancer [after failing oxalplatin and/or irinotecan regimens]		--′′--
--′′--	Vorinostat (Zolinza)	Chemotherapeutic	Histone deacetylase (HDAC) inhibitor, promoting apoptotic cell death and cell cycle arrest in G1, G2/M	1,2		Refractory cutaneous T cell lymphoma	--′′--

* MAID: Mesna, Doxorubicin, Ifosfamide, Dacarbazine; ′′ABVD: Adriamycin (Doxorubicin), Bleomycin, Vinblastine, Dacarbazine.

**Table 2:** Chemotherapeutic drugs.

Alkylating Agents	Drugs	Mechanism of Anti-Tumor or Action
Nitrogen mustards:	busulfan, chlorambucil, melplatin	Proliferation block by creating inter- or intra-strand cross links in DNA, or
Platinum based:	cisplatin, carboplatin, oxalplatin	causing DNA base mispair, thereby
Qxazaphosphorines:	cyclophosphamide, ifosfamide	preventing strand separation during cell cycle progression
Hydrazine		
Carmustine		
Antimetabolites		
Purine analogs:	6-mercaptopurine, azathioprine, cladribine	Proliferation or cell cycle block by:
Purine antagonists:	fludarabine	interference with biosynthetic pathways,
Pyrimidine antagonists:	cytarabine, 5-fluorouracil (5-FU), gemcitabine, capecitabine	disturbance of DNA/RNA formation,
Antifolates	methothrexate, pemetrexed, pralatrexate	causing DNA strand breaks, and
Inhibitors of ribonucleotide reductase	hydroxyurea	Incorporation of false analogues. These events ultimately can trigger apoptosis.
**Mitotic Spindle Poisons (Mitosis Poisons)**		
Taxans:	docetaxel, paclitaxel, cabazitaxel	Preventing depolymerization of mitotic spindle by stabilizing GDP-bound tubulin in microtubule.
Vinca alkaloids:	vincristine, vinblastine, vinorelbine, vindesine, vinflunine	Preventing mitotic spindle formation by inhibition of tubulin polymerization.
**Others**		
Antibiotics:	bleomycin, actinomycin D, anthracyclines	Intercalates into DNA stopping transcription.
Proteasome inhibitors	bortezomib	Apoptotic cell death.
Tyrosine kinase inhibitors:	imatinib, erlotinib	Affecting multiple signaling pathways.
Enzymes	l-asparaginase	Deregulates normal metabolism.
Topoisomerase I; inhibitors:	irinotecan, topotecan	DNA strand breaks during replication and
Topoisomerase II; inhibitors:	etoposide, anthracyclines: doxorubicin,	causing cell cycle block, and indirectly apoptosis.

**Table 3:** Historical perspective of anticancer drugs: Part 2. 2008–2014.

Year of Approval	Drug (Therapy)	Category	Mode of Action	Targeted Hallmark	First Indications (Current)		
					Solid Tumors	Blood Borne	Institution or Country
2008	Oncophage	Therapeutic Vaccline	Bolstering anticancer immune response by autologous tumor-deriv heat shock protein gpg6	10	Renal cell carcinoma		Russia
2009	Cervarix	Therapeutic Vaccline	Vaccine against two types of HPV (16 and 18)	10	Prevention of cervical cancer and other cancers in the reproductivr organs		FDA
2011	Sipuleucel-T (Provenge)	Therapeutic Vaccline (autologous cellular immunotherapy)	Bolstering anti prostate cancer adaptive immune response	10	Castration resistant prostate cancer		FDA
--′′--	Lpilimumab (Yervuy)	Immunotherapeutic/Biologic	Immune checkpoint inhibitor of CTLA-4	10	Melanoma [matastatic]		EMA, TGA, FDA
--′′--	Vemurafenib (Zelboraf)	Chemotherapeutic	Inhibits proliferation without growth factors by inhibiting mutated BRAF serine-threonine kinase	1	Advanced melanoma with BRAF V600 mutation		FDA
--′′--	Brentuximab vedotin (Adcetris)	Drug-kibked biologic	Cytot10oxic ag10ent-linked10 chimer10ic mouse/hum10an anti-huna1n CD301,2	1,2		Hodgkin lymphoma, anaplastic large cell lymphoma; (cutaneous T cell lymphoma, peripheral T cell lymphoma)	FDA (EMA in 2012)
--′′--	Peginterferon alfa-2b (Sylatron)	Biologic	Cytokine, stimulates killing of tumor cells	10	Melanoma		FDA
2012	Carfilzomib (Kyprolis)	Chemotherapeutic	Irreversible proteasome inhibitor, cell cycle block, apoptosis	2	Relapsed or refractory multiple melanoma		FDA
2013	Pomalidomid (Pomalyst)	Chemotherapeutic (Thalidomide analogue)	An immunomoduatory drug, targets, protein cereblon; inhibits COX2, inhibits angiogenesis, induces apoptosis via G1 arrest	1,2,8	Relapsed or refractory multiple melanoma		FDA
2014	Blinatumomab (Blincyto)	Biologic	moAb, a bispecific T-cell engager (BiTE); CD 19 poditive cancers are killed by cytotoxic T cells	10		B cell acute lymphoblastic leukemia [ALL]	FDA
--′′--	Tisagenlecleucel (Kymriah)	CAR T cell immunotherapy	Targeting the CD 19 receptor on cancer cells	1,2		B-ALL, (EMA in 2016, relapsed or refractory diffuse large B cell lymphoma; FDA in 2018)	EMA (FDA in 2017)
--′′--	Ramucriumab (Cyramaza)	Biologic	moAB that blocks interaction of VEGFR2 with ligands, inhibiting angiogenesis	4	Advanced stomach cancer and gastroesophageal junction adenocarcinoma after prior therapy		FDA
--′′--	Pembrolizumab (Keytruda) and nivolumab (Opdivo)	Immunotherapeutic/Biologic	Immune checkpoint inhibitor of PD-1	10	Not resectable melanoma; with ipilimumab-numerous indications (see in Table 5)		EMA, FDA, TGA and Japan

**Table 4:** Historical perspective of anticancer drugs: Part 3. 2015–2020.

Year of Approval	Drug (Therapy)	Category	Mode of Action	Targeted Hallmark	First Indications (Current)		
					Solid Tumors	Blood Borne	Institution or Country
2015	Panabinostat (Farydak)	Chemotherapeutic	Histone deacetylase inhibitor, promoting cell death and cell cycle arrest	2		Multiple myeloma, relapsed or refractory, in those previously treated with bortezomib and lenalidomide or thalidomide	FDA
--′′--	Palbociclib (Ibrance)	Chemotherapeutic/small molecules inhibitor	Inhibitor of cyclin-dependent kinase (CDK) 4 and 6	1	With an aromatase inhibitors as initial therapy of postmenopausal, HR-positive, HER2-negative advanced or metastatic breast cancer		FDA
2017	Atezolizumab (Tecentriq)	Immunotherapeutic/biologic	Anti-PD-L1 checkponit inhibitor	10	Metastatic, chemotherapy-resistant non-small cell lung cancer [NSCLC]		FDA
--′′--	Olaratumab (Lartruvo)	Immunotherapeutic/biologic	Antibody against the PDGFRα	1	Soft tissue sarcoma [STS], provided ineffective surgery and radiation therapy (withdrawn in 2019, EMA and FDA)		EMA, FDA
--′′--	Gemtuzumab ozogamicin (Mylotarg)	Drug-linked biologic	Anti-CD33 conjugated to toxin	1,2		CD33-positive acute myeloid leukermia [AML]	FDA
--′′--	Durvalumab (Imfinzi) and avelumab (Bavencio)	Immunotherapeutic/biologic	Anti-PD-1/PD-L1 checkpoint inhibitors	10	Advanced bladder cancer		FDA
--′′--	Axicabtagene ciloleucel (Yescarta)	CAR T cell immunotherapy	Targeting the CD19 receptor on cancer cells	1,2		Several types non-Hodgkin large B cell lymphomas refractory or twice relapsed	FDA
--′′--	Ribociclib (Kisqali)	Chemotherapeutic/ small molecules inhibitor	Cyclin dependent kinase inhibitor (CDKi)	1	With an aromatase inhibitors as initial therapy of postmenopausal, HR-positive, HER2-negative advanced or metastatic breast cancer		FDA
--′′--	Abemaciclib (Verzenio)	Chemotherapeutic/ small molecules inhibitor	Inhibitor of cyclin-dependent kinase (CDK) 4 and 6	1	With an aromatase inhibitors as initial therapy of postmenopausal, HR-positive, HER2-negative advanced or metastatic breast cancer		FDA
2018	Cemiplimab (Libtayo)	Immunotherapeutic/biologic	moAB, anti-PD-1 checkpoint inhibitor	10	Metastatic cutaneous squamous cell carcinoma [CSCC] or lacally advanced CSCC who are not candidates for curative surgery or surative radiation		FDA (EMA in 2019, TGA in 2020)
2019	Pexidartinib (Turalio)	Small molecule immunomodulator (chemotherapeutic)	Targeting the cytokine CSF-1 receptor pathway	1	Symptomatic tenosynovial giant cell tumor		FDA
--′′--	Venetoclax (Venclexta, Venclyto)	Chemotherapeutic/ small molecules inhibitor	Targeting Bcl-2	2		CLL, small lymphocytic lymphoma [SLL], AML	FDA (EMA in 2020)
2020	Brexucabtagene autoleucel (Tecartus)	CAR T cell immunotherapy	Targeting the CD19 receptor on cancer cells	1,2		Relapsed or refractory Mantle cell lymphoma	FDA
--′′--	Gardasil 9	Prophylactic vaccine	Anti-HPV (type 6, 11, 16 and 18)	10	Head and neck HPV-related cancer prevention		EMA, FDA

**Table 5:** Current therapeutic indications of immune checkpoint inhibitors.

Therapy	Mode of Action	Approval	Indications
Ipilimumab (Yervoy)	Inhibitor of CTLA-4	Since 2011	Melanoma (metastatic)
Nivolumab (Opdivo)	Inhibitor of PD-1	Since 2014	(1) surgically inoperative melanoma;
			(2) relapsed colorectal cancer that is characterized by high microsatellite instability (MSI-hi),
			(3) gastric cancer (The Pharmaceuticals and Medical Devices Agency (PMDA) of Japan),
			(4) advanced liver cancer that has been previously treated with sorafenib;
		Since 2018	(5) mesothelioma (PMDA);
		Since 2020	(6) unresectable advanced or recurrent esophageal cancer that has progressed following chemotherapy (PMDA),
			(7) unresectable advanced, recurrent or metastatic esophageal squamous cell carcinoma after previous fluoropyrimidine- and platinum-based chemotherapy.
Pembrolizumab (Keytruda)	Inhibitor of PD-1	Since 2014	(1) surgically inoperative melanoma;
		Since 2017	(2) advanced non-small cell lung cancer (NSCLC, first line),
			(3) bladder cancer (first line),
			(4) all metastatic solid tumor types classified as MSI-hi (high microsatellite instability) or dMMR (deficient DNA mismatch repair) (second line),
			(5) advanced recurrent cancer of the stomach and gastroesophageal junction;
		Since 2018	(6) patients with cervical cancer expressing PD-L1 that is metastatic or has recurred after previous chemotherapy treatment,
			(7) adult and pediatric patients with primary mediastinal large B-cell lymphoma (PMBCL) that is refractory or has relapsed after two or more prior systemic treatments,
			(8) advanced, treatment-resistant hepatocellular carcinoma, the most common type of liver cancer;
		Since 2019	(9) stage III non-small cell lung cancer (NSCLC) that is PD-L1-positive and is not amenable to surgery or chemo-radiation treatment (first-line),
			(10) advanced esophageal squamous cell cancer,
			(11) advanced endometrial carcinoma in patients with disease progression following prior systemic therapy but are ineligible for surgery or radiation,
		Since 2020	(12) advanced endometrial carcinoma in patients with disease progression following prior systemic therapy but are ineligible for surgery or radiation,
			(13) unresectable or metastatic microsatellite instability-high (MSI-H) or mismatch repair deficient (dMMR) colorectal cancer (first line),
			(14) recurrent or metastatic cutaneous squamous cell carcinoma that is not curable by surgery or radiation,
			(15) unresectable or metastatic tumor mutational burden-high solid tumors, which have progressed and have no satisfactory alternative treatment options.
Durvalumab (Imfinzi)	anti-PD-L1 inhibitor	Since 2014	(1) advanced bladder cancer,
		Since 2018	(2) unresectable, stage III non-small cell lung cancer (NSCLC) that hasn’t progressed after prior chemo-radiation treatment;
		Since 2020	(3) extensive-stage small cell lung cancer (ES-SCLC) in combination with standard-of-care chemotherapy (as a first line).
Avelumab (Bavencio)	a PD-L1 inhibitor	Since 2014	(1) advanced bladder cancer,
		Since 2017	(2) for the treatment of Merkel cell carcinoma (EMA),
		Since 2020	(3) for maintenance treatment of patients with locally advanced or metastatic urothelial carcinoma that has not progressed with first-line platinum-based chemotherapy.
Atezolizumab (Tecentriq)	anti-PD-L1 inhibitor	Since 2014	(1) metastatic, chemotherapy-resistant NSCLC,
		Since 2019	(2) unresectable (inoperable) or metastatic triple-negative breast cancer that also expresses PD-L1, (in combination with chemotherapy, as a first line).
			(3) small cell lung cancer-ES-SCLC, (in combination with chemotherapy, as a first line).
			(4) metastatic non-small cell lung cancer-nonsquamous NSCLC without EGFR or ALK molecular aberrations, (in combination with chemotherapy, as a first line).
		Since 2020	(5) BRAF V600 mutation-positive advanced melanoma (in combination with cobimetinib and vemurafenib).
Cemiplimab (Libtayo)	anti-PD-1 inhibitor	Since 2018	cutaneous squamous cell carcinoma, metastatic or locally advanced
Nivolumab	inhibitor of PD-1	Since 2018	(1) melanoma (PMDA),
plus			(2) advanced renal cell carcinoma, the most common form of kidney cancer (FDA, EMA)
ipilimumab	inhibitor of CTLA-4		(3) relapsed or refractory colorectal cancer characterized by high microsatellite instability (MSI-hi) or deficient DNA mismatch repair (dMMR) (FDA).
		Since 2020	(4) advanced hepatocellular carcinoma, the most common form of liver cancer, in patients who have previously been treated with sorafenib.(FDA)
			(5) metastatic non-small cell lung cancer (NSCLC) that expresses PD-L1 and does not possess mutations in the EGFR or ALK genes. A triple combination comprising nivolumab, ipilimumab and platinum-doublet chemotherapy was approved (FDA) as a first-line therapy for the same indication including recurrent NSCLC.
Atezolizumab	anti-PD-L1 inhibitor	Since 2020	previously untreated hepatocellular carcinoma.
plus			
bevacizumab	anti-VEGF Ab		
